# Strike 3 … Out! Investigating Pre-Game Moods, Performance, and Mental Health of Softball Umpires

**DOI:** 10.3390/sports12020050

**Published:** 2024-02-02

**Authors:** Ronald J. Houison, Andrea Lamont-Mills, Michael Kotiw, Peter C. Terry

**Affiliations:** 1 School of Psychology and Wellbeing, University of Southern Queensland, Toowoomba 4350, Australia; peter.terry@unisq.edu.au; 2 Academic Affairs Administration, University of Southern Queensland, Ipswich 4305, Australia; andrea.lamont-mills@unisq.edu.au; 3 School of Health and Medical Sciences, University of Southern Queensland, Ipswich 4305, Australia; mike.kotiw@unisq.edu.au

**Keywords:** mood profile, sports official, Brunel Mood Scale, softball, umpire, wellbeing

## Abstract

Mood research in sports typically focuses on athletes, with sports officials being largely overlooked. In the current study, mood profiling was used to determine if softball umpires reported an identifiable and consistent mood profile and if mood was predictive of umpiring performance and/or reflective of positive mental health. Eleven male and five female participants aged 25–68 years (M = 48.5 ± 15.5 years) each completed the Brunel Mood Scale on multiple occasions prior to officiating games at the 2020 U18 National Softball Championships. A total of 185 mood profiles were analysed. Performance was assessed using Softball Australia’s official umpire assessment tool. Overall, participants reported an iceberg mood profile, which tends to be associated with positive mental health and good performance. Umpiring performances (pass/fail) were correctly classified in 75.0% of cases from tension, depression, and confusion scores (*p* = 0.003). Participant sex explained 25.7% of the variance in mood scores (*p* < 0.001); age, 25.8% of the variance (*p* < 0.001); position on the diamond, 10.5% of the variance (*p* = 0.003); and accreditation level, 14.3% of the variance (*p* < 0.001). Australian softball umpires typically reported mood profiles associated with positive mental health, and none reported profiles associated with risk of mental ill-health.

## 1. Introduction

Mood profiling has been used in sports since the 1970s, when Morgan [1] hypothesised that mood profiles may be predictive of athletic success. Most sports performance research, including mood research, has focused on athletes and coaches [2,3]. However, there is another group of participants who have a significant impact on sports, the officials. Sports officials (i.e., referees, umpires, or judges) are expected to perform to a high standard under the pressure of expectation from participants, fans, and sponsors in complex situations, which often leads to heightened physical and cognitive load [4].

Moods have been conceptualised as a collection of feelings that often lack identifiable triggering events and are less intense and tend to persist longer than emotions [5]. Moods can be described as bi-dimensional, having valence that varies from positive to negative and arousal that varies from activation to deactivation [6]. The mood of individuals is typically assessed using a self-report scale such as the Profile of Mood States (POMS) [7] or its derivative, the Brunel Mood Scale (BRUMS) [8,9]. Mood profiling is a process in which an individual’s mood scores are plotted against test norms and presented graphically [10]. This profile can then be used to identify commonly occurring patterns of mood responses and to assess relationships between moods, performance, and psychological wellbeing [10,11,12].

Morgan [13,14] proposed that athletic success was strongly associated with positive mental health and found POMS scores to be predictive of athletic success. Morgan [1] reported that, when plotted against normative data, the mood profiles of athletes chosen to represent the USA in wrestling at the 1972 and 1976 Olympic games resembled an iceberg, with the score for vigour above the mean population score of 50 and the scores for tension, depression, anger, fatigue, and confusion below the mean. The iceberg profile was the first mood profile to be used to predict successful athletic performance. The subsequent development of the BRUMS specifically for the purpose of assessing mood states in sport-related contexts using tables of normative values based on athletic samples [8,9] enhanced the utility of mood profiling in sport.

Mood profiling has been applied in a variety of contexts, such as screening for the risk of posttraumatic stress disorder in a military population [15], monitoring the wellbeing of cardiac rehabilitation patients [16], and identifying increased risk of psychopathology during COVID-19 restrictions [17]. However, the use of mood profiling remains most prevalent in sporting contexts. In addition to the iceberg profile, several additional mood profiles have been documented. The Everest profile [10], for example, is represented by near maximum scores for vigour and near minimum scores for depression, tension, confusion, anger, and fatigue, and it is proposed to be a good indicator of superior sports performance. Conversely, the inverse iceberg profile is characterised by below-average scores for vigour and above-average scores for depression, tension, confusion, anger, and fatigue, and it is seen as an indicator of poor sports performance and the risk of impaired mental health [18,19]. Four further profiles were identified in the general population; the inverse Everest, shark fin, submerged, and surface profiles [20]. These four profiles plus the iceberg and inverse iceberg profiles have been identified in multiple cultural and language contexts, using sport and community samples [21,22].

Officials are key participants in team sports [4,23]. They can be classified as interactors (e.g., referees or umpires), reactors (e.g., tennis line judges), or monitors (e.g., dressage or gymnastics judges) with the classification based on the number of athletes to be monitored and the interaction required with athletes [24]. The developmental pathways and motivations of sport participants who later became officials were explored by Hancock and his associates [25], who reported that sport participants typically began and continued officiating for intrinsic, sport, and social reasons. Many athletes choose to officiate to prolong their career in the sport, because of encouragement from significant social and family members, or to participate at higher levels than they had as an athlete.

Like athletes, elite-level officials are expected to acquire and apply in-depth knowledge about their role in complex situations and adapt to changing needs as they progress in their sport, such as increased physical demands, greater pressure to perform, changing contexts, and changing athlete playing strategies [4]. Officials are often treated poorly with attitudes that range from indifference to hostility, death threats, and violence [26,27]. Consequentially, officials require considerable resilience [26], a quality found in individuals with robust mental health. The combination of increased performance expectations, scrutiny, and criticism suggested a need for research into the mental health of sports officials. Given that mood profiling serves a dual role of providing both an indicator of mental health status [11,12] and a predictor of performance in sports [28,29], research using mood profiling among sports officials appears to be appropriate, and Australian softball umpires (interactor-class officials) were selected as the participants in this study.

Softball umpires are appointed to games in roles broadly categorised as plate or field umpires. Plate umpires are initially positioned behind the home plate and a player known as the “catcher”. The plate umpire is required to judge whether each unhit pitch (a ball thrown with an underarm action by a player called the “pitcher” to the catcher) is either a “strike” or a “ball”. A batter who accumulates three strikes is called out; a batter who accumulates four balls is awarded first base. The umpire behind the plate adjudicates on relevant plays (such as batter and runners being safe or out) and manages issues beyond plays, such as deciding rule interpretations and player substitutions. Field umpires are positioned initially at bases (i.e., first, second, or third base) or in the outfield and adjudicate on plays in their vicinity, such as a runner being safe or out, or a batter being out after a catch was completed [30]. A more detailed overview of softball can be found at https://en.wikipedia.org/wiki/Softball (accessed on 9 December 2023).

Previous research has identified significant sex and age differences in reported mood. Terry and his associates [31] reported that females scored higher than males in tension, fatigue, and confusion dimensions, and males scored higher than females in anger and vigour. No sex differences were found for depression. Various age group differences were reported. Vigour scores for participants aged 46–55 years and aged 56+ years were higher than those for participants aged 25–45 years. Fatigue and confusion scores were lower for participants aged 56+ years than for participants aged 18–24 years. The 46–55 years group scored higher in depression than those aged 18–24 years [31]. The influence of these demographic variables will similarly be examined in this study. Further, the influence on mood responses of key situational variables, novel and specific to softball umpires, is of considerable interest; these are their role as a plate or field umpire and the umpire accreditation level they have achieved.

The extensive literature on the impact of mood on athletic performance and the need for further psychological research involving sports officials [23] prompted the present study, which was the first to examine mood factors and the performance of softball umpires. Based on prior findings [11,12,28,29], it was hypothesised that participants would report a consistent mood profile synonymous with positive mental health (H1) and that mood would be predictive of umpiring performance (H2). Also, based on previous research [31], it was hypothesised that male umpires would report more positive moods than female umpires (H3) and that older umpires would report more positive moods than younger umpires (H4). Although the effects on mood of umpire role and accreditation level have not previously been investigated, it was hypothesised (H5) that plate umpires would report higher tension scores than field umpires due to the greater cognitive demands of the role, and (H6) that umpires achieving higher accreditation levels would report more positive mood than those at lower accreditation levels, due to greater experience in the role. Therefore, the aims of the study were (a) to investigate the mood profiles of Australian softball umpires officiating at a national championship in relation to umpiring performance and mental health status and (b) to assess the influence of participant sex, age, umpire role, and accreditation level.

## 2. Materials and Methods

### 2.1. Participants

Eleven male and five female participants aged 25–68 years old (females M = 38.06 ± 12.73 years, males M = 54.07 ± 13.83 years) were recruited from the 21 officials appointed to the Softball Australia U18 Women’s and U18 Men’s 2020 national softball championships (*N* = 16, M = 48.5 ± 15.5 years). This sample represented 76.2% of the population of interest. Seven participants officiated at the U18 Men’s Championship (8–14 December 2019), seven participants officiated at the U18 Women’s Championship (19–25 January 2020), and two participants officiated at both championships. The participants’ umpiring accreditation level ranged from Level 4 to Level 8 (M = 5.9 ± 0.9). Australian umpire accreditation ranges from Level 1 to Level 8, with a higher numbered level being more highly qualified. Level 1 requires theory exam passes and no formal assessment. Levels 2–4 are assessed via theory exams and practical exams at state competitions, and Levels 5–6 are assessed via theory exams and practical exams at national competitions. Levels 7–8 are awarded for contributions to the umpiring program [32], which usually involves achieving international accreditation from the World Baseball Softball Confederation (WBSC) [33] and having completed appointments to officiate at WBSC world championships. Participant umpiring experience ranged from 7 to 35 years (M = 20.3 ± 8.2 years). Participation in the study was voluntary, and no incentives were provided.

### 2.2. Measures

Pre-game mood was measured using the BRUMS [8,9]. This questionnaire consists of 24 items with 4 items in each of the six subscales of anger, confusion, depression, fatigue, tension, and vigour. Respondents indicated the extent to which they were experiencing each mood item on a 5-point Likert scale ranging from 0 (not at all) to 4 (extremely) in response to the question “How do you feel right now”. Responses to each subscale item were summed to give a subscale score ranging from 0 to 16 where higher scores indicate higher levels of the described mood dimension [8,9]. The psychometric properties of the BRUMS were evaluated using multi-sample confirmatory factor analysis across samples of adult students and athletes, young athletes, and schoolchildren, which supported the configural, metric, scalar, and residual invariance of the measurement model [8,9]. Cronbach alpha coefficients ranging from 0.74 to 0.90 for the six subscales have been reported, supporting the internal consistency of the BRUMS subscales [8,9]. In the present study, the Cronbach alpha coefficients for the six subscales were anger = 0.75, confusion = 0.72, depression = 0.76, fatigue = 0.79, tension = 0.86, and vigour = 0.82.

Participant performance was evaluated in each game using Softball Australia’s umpire assessment tool [34]. This is a standard tool used to assess the performance of softball umpires in Australia and has been used since the 1990s. Assessments were made by members of the championship umpire management teams, who were appointed by Softball Australia and were independent of the research team. The assessment process is part of the normal umpiring performance assessment that occurs at each championship. The management teams each consisted of three assessors, all with international-level umpiring qualification and experience and accredited by Softball Australia as senior assessors. There are 23 performance categories assessed and scored under the following five areas of umpiring: General, Game Control, Judgement and Rules, Positioning and Calls, and Plate Work. Marks are assigned in each category on a 5-point scale ranging from 1 (The candidate fails to comply with more than 4 requirements of this category) to 5 (The umpire does everything that the category requires but at times displays exceptional umpiring ability and skills), with 4 (The umpire complies with the minimum requirements in each category) being the default score. Category marks were reduced by 1 from the default score of 4 on an “errors-made” basis. After the first error is recorded, participants are penalised for every second subsequent error (i.e., on the first, third, and fifth errors). A category score of 5 is only achievable when no errors are attributed to the category and the participant is subjectively judged to have performed at an exceptional standard.

To obtain the participant’s performance score for each game, raw score totals in each section were converted to scaled marks via the Candidate Umpire Conversion Chart and then totalled. Higher scores signify better performance with game pass marks assigned to each accreditation level and on-diamond position/role (i.e., plate umpire or base/field umpire): Level 4: Plate = 80, base = 61; Level 5–8: Plate = 82, base = 61 [34].

Observation data were collected by senior officials for the purpose of assessing the performance of the participants. Data consisted of the name of the participant, position on the diamond, assessment categories to which observations related, details of the observed behaviour of the participant on the diamond, and a flag to indicate whether the observation is scored as a positive or negative behaviour. These data were used in conjunction with the umpire assessment tool to assess participants’ performance.

### 2.3. Procedure

The study was conducted over two one-week periods, 7–14 December 2019 and 18–25 January 2020. Officials appointed to officiate at the 2020 U18 National Softball Championships were invited to participate in the research by Softball Australia via email prior to the commencement of the championships. Each participant provided written consent and demographic information including age, sex, accreditation level, and years of experience at umpire meetings prior to the commencement of the championships.

Participants completed a paper version of the BRUMS [8,9] before each game they officiated. The questionnaire was provided to participants 45 min prior to the scheduled time for the game to commence and collected by the first author no later than 30 min prior to game time except when participants were appointed to consecutive games and the time between games was less than 60 min. In these cases, the questionnaire was completed no less than 20 min before the commencement of the second game to be officiated. Participants completed the survey in the officials’ change rooms, a relatively quiet and private environment. Each participant officiated in between 7 and 13 games, except for one part-time participant who officiated in only two games. The total number of completions of the BRUMS was 185.

The most appropriate normative means against which to compare the mood profiles of officials depends on whether they are regarded as athletes or non-athletes. For example, the role of the softball umpire is sedentary compared to more active officials, such as Australian Football League (AFL) umpires who can travel more than 11 km during a game [35]. Softball umpires, on the other hand, spend most of the game relatively stationary with occasional running for a distance up to 120 feet (base and field umpires). The plate umpire is more active and will squat and hold for a few seconds for every pitch thrown and run up to 60 feet [30]. Hence, it was decided that softball umpires should, for the purposes of this research, be considered as non-athletes, and hence, tables of normative data for the BRUMS specific to male and female non-athletes [11] were used to standardise the mood scores of the participants. An Excel spreadsheet containing details of the assessment category and assessable situation was created and maintained at each championship. Observations of each participant’s performance in every game were documented by members of the umpire management team in accordance with Softball Australia’s procedures [36]. These spreadsheets were provided to the research team by Softball Australia’s Umpire-in-Chief (Development), and the first author calculated the participants’ performance in each game using Softball Australia’s umpire assessment tool [34].

### 2.4. Ethics

This study was conducted under ethics approval #H19REA271 granted by the University of Southern Queensland human ethics committee. Recruitment of participants officiating at the U18 Men’s Championship was conducted and written consent was obtained at the umpire meeting on 8 December 2019. Recruitment of participants officiating at the U18 Women’s Championship was conducted and written consent was obtained at the umpire meetings on 18–19 January 2020. As part of the informed consent process, participants were allocated a randomly generated 5-digit number (random.org accessed on 2 December 2019) which was used as a unique ID to ensure the confidentiality of participant data. All participants were 18+ years of age when consent was provided.

### 2.5. Statistical Analysis

SPSS for Windows, Version 29, IBM Corporation, Armonk, NY, USA [37], was used to conduct all statistical analyses. Descriptive statistics were first calculated for all mood scores and compared to population means. Next, a stepwise regression analysis was performed to predict umpiring performance scores from the six pre-performance mood scores, followed by a discriminant function analysis to determine the percentage of umpiring performances that could be correctly classified as pass or fail from mood scores. Multivariate analysis of variance (MANOVA) was used to compare mood scores by performance result (pass/fail), participant sex, participant age, participant position on the diamond, and umpire accreditation level. One of the assumptions underlying MANOVA procedures is that all measures are independent. Although we combined multiple mood profiles from the same participants into a single dataset, thereby treating them as independent data points, MANOVA has been shown to be robust even when underlying assumptions are not met [38]. Follow-up univariate tests were performed using a Bonferroni-adjusted alpha level of *p* < 0.008 (i.e., *p* < 0.05 divided by 6) due to having six dependent variables: tension, depression, anger, vigour, fatigue, and confusion. Effect sizes in the form of Cohen’s d were interpreted as small (0.20), moderate (0.50), or large (≥0.80) [39], and effect sizes in the form of partial eta-squared were interpreted as small (0.01), moderate (0.06), or large (0.14) [39].

### 2.6. Data Screening

Cases were screened for implausible response patterns (e.g., scoring 0 or 16 on all subscales) and none were identified. No missing values were detected. Two cases violated the timing criteria for completion of the BRUMS (between 45 and 30 min prior to game commencement) and were discarded. In the first case, the BRUMS was completed 25 min before a game, and in the second, the BRUMS was completed more than 45 min before a game due to the game’s delayed commencement. Significant deviation from univariate normality was evident for some subscales, with positive skewness ranging from 2.60 to 10.65 for tension, depression, anger, and confusion scores. Kurtosis values were high, ranging from 4.63 to 126.30 for all subscales except vigour. Such non-normality was expected, given that the distribution of negative mood scores typically trends towards the lower end of the scale [8,9] and high scores on mood subscales, which are of particular interest, are often identified as outliers. Detailed scrutiny of the dataset showed no evidence of response bias in the form of acquiescent, extreme, or straight-line responding [40,41], and therefore, all cases were retained and no data transformations occurred.

## 3. Results

### 3.1. Group Mood Profile

**Figure 1:**
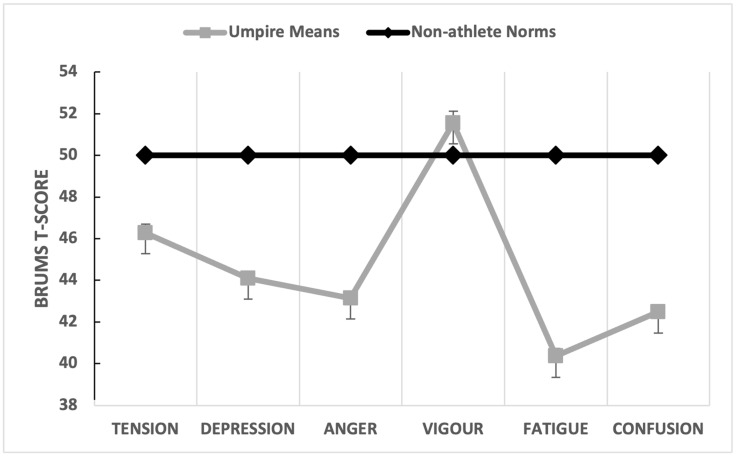
Graphical representation of mean mood profile for umpires (*N* = 185) compared to non-athlete norms [11].

The first step in the data analysis was to assess the collective mean mood profile for all umpires across all games. BRUMS scores were converted to standard scores (T-scores) [11] (see Table 1). As a group, the umpires reported a mood profile associated with positive mental health and good performance, with mean scores for tension, depression, anger, fatigue, and confusion significantly below population means. Effect sizes (d) were very large for depression, anger, fatigue, and confusion; moderate for tension; and small for vigour. An iceberg profile, albeit with the mean vigour score only just above the population norm, emerged when the group means for each mood dimension were plotted graphically (Figure 1).

### 3.2. Classification of Umpiring Performance from Mood

When umpiring performance was dichotomised into pass (n = 154) and fail (n = 30) categories, a discriminant function analysis (Wilks’ *λ* = 0.894, *p* = 0.003) showed that performance could be correctly classified as pass/fail from pre-performance mood scores with 73.4% accuracy. Correct classifications improved to 75.0% when only the tension, depression, and confusion scores were included in the analysis.

### 3.3. Mood Scores by Grouping Variables

#### 3.3.1. Effect of Umpire Performance

**Figure 2:**
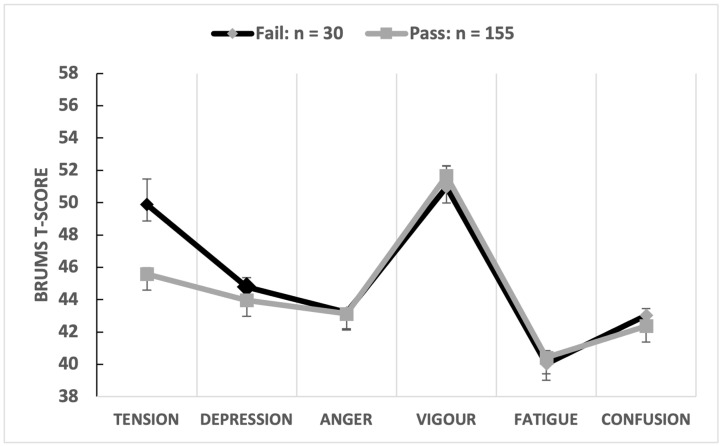
Graphical representation of mood profiles by umpiring performance.

The multivariate comparison was significant (Wilks’ λ = 0.894, *p* = 0.003, partial η^2^ = 0.106), with umpiring performance (pass/fail) explaining 10.6% of the overall variance in mood scores, a moderate-to-large effect. Univariate comparisons showed that tension scores were significantly higher in games scored as fail (Table 2). The game result explained 7.2% of the variance in tension scores with a moderate-to-large effect size (d = 0.73). Some subscale scores (notably for anger and fatigue) were reported at uniformly low levels. For example, 184 cases (99.5%) reported anger T-scores in the range 43–46, equating to a raw score of 0 or 1. This minimal range of scores limited the predictive relationship of anger scores on performance and all other variables of interest. An iceberg profile was reported for both pass and fail games when the means of the mood dimensions were graphed separately (Figure 2).

#### 3.3.2. Effect of Participant Sex

**Figure 3:**
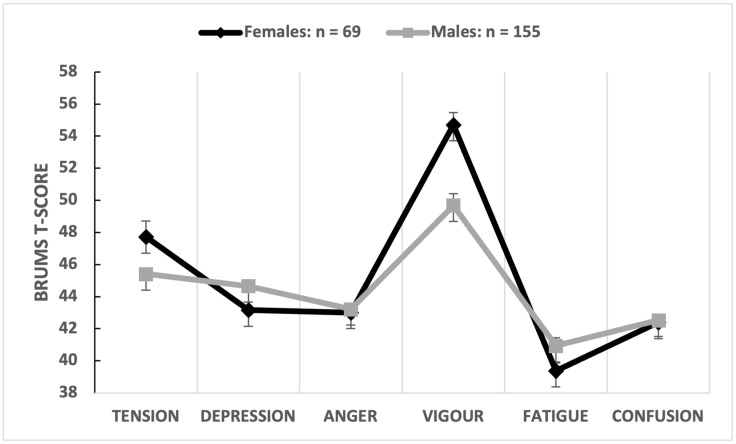
Graphical representation of mean mood profiles by sex of umpire.

The multivariate comparison was significant (Wilks’ λ = 0.743, *p* < 0.001, partial η^2^ = 0.257), with the sex of participants explaining 25.7% of the overall variance in mood scores, a large effect. Univariate comparisons showed that depression scores were significantly higher for male participants whereas vigour scores were significantly higher for female participants (Table 3). The sex of the participant explained 12.5% of the variance in depression scores with a moderate-to-large effect size (d = 0.73) and explained 9.9% of the variance in vigour scores with a moderate effect size (d = 0.65). An iceberg profile for females and a submerged profile for males resulted when the means of the mood dimensions were graphed separately (Figure 3).

#### 3.3.3. Effect of Participant Age

**Figure 4:**
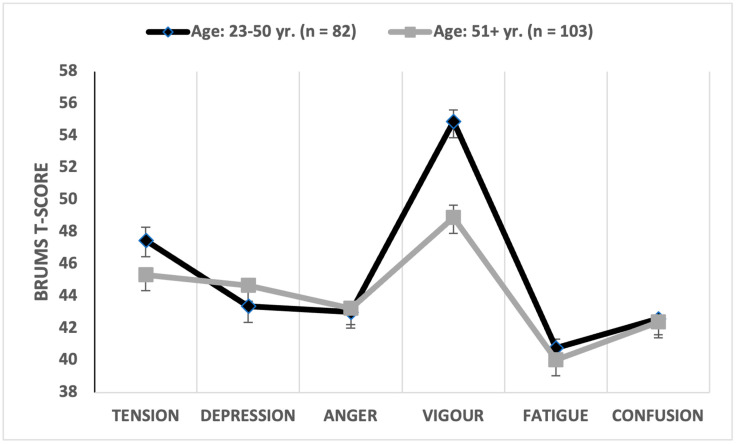
Graphical representation of mean mood profiles by age.

Given that age has previously been shown to influence mood [31], participants were grouped on the basis of a median split to create two approximately equal-sized groups (25–50 years, 51+ years). The multivariate comparison was significant (Wilks’ λ = 0.742, *p* < 0.001, partial η^2^ = 0.258), with age explaining 25.8% of the overall variance in mood scores, a large effect. Univariate comparisons showed that vigour scores were significantly higher and depression scores significantly lower for participants aged 25–50 years old than for participants aged 51+ years old (Table 4). The age group of the participant explained 10.1% of the variance in depression scores with a medium effect size (d = 0.69) and 14.9% of the variance in vigour scores with a moderate-to-large effect size (d = 0.78). When the means of the mood dimensions were graphed separately, an iceberg profile was reported for participants aged 23–50 years old, and a submerged profile was reported for participants aged 51+ years old (Figure 4).

#### 3.3.4. Effect of Field Position

**Figure 5:**
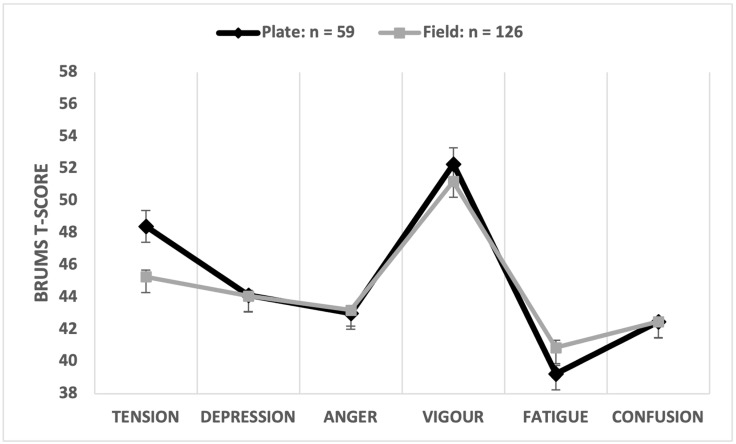
Graphical representation of mean mood profiles by umpire field position.

The multivariate comparison was significant (Wilks’ λ = 0.895, *p* = 0.003, partial η^2^ = 0.105), with field position explaining 10.5% of the overall variance in mood scores, a moderate-to-large effect. Univariate comparisons showed that tension scores were significantly higher for plate umpires (Table 5). The field position of the participant explained 6.1% of the variance in tension scores with a moderate effect size (d = 0.53). Iceberg profiles were evident for both plate and field umpires (Figure 5).

#### 3.3.5. Effect of Accreditation Level

**Figure 6:**
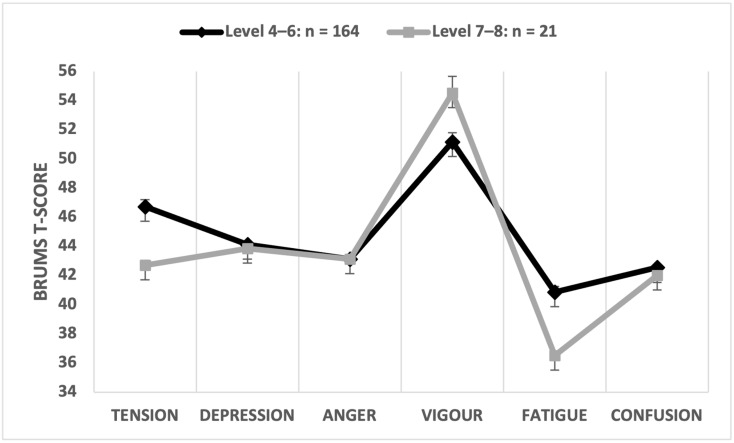
Graphical representation of mean mood profiles by accreditation level.

Participants were allocated to one of two groups based on the umpire holding an Australian national qualification (Levels 4–6) or holding an international-level qualification and having officiated at a world championship (Level 7–8). The multivariate comparison was significant (Wilks’ λ = 0.857, *p* < 0.001, partial η^2^ = 0.143), with accreditation level explaining 14.3% of the overall variance in mood scores, a large effect. Univariate comparisons showed that tension and fatigue scores were significantly higher for Level 4–6 participants (Table 6). The accreditation level of the participant explained 4.7% of the variance in tension scores with a very large effect size (d = 1.91) and explained 7.8% of the variance in fatigue scores with a large effect size (d = 0.88). An iceberg profile was reported for both accreditation groups (Figure 6).

## 4. Discussion

The present study took the approach of assessing the mood profiles of softball umpires, in a similar manner to studies of athlete mood profiles, with the aim of determining if their mood profiles were reflective of positive mental health and/or were predictive of umpiring performance. The study also assessed the effects of the sex and age of the umpire, position on the diamond, and accreditation level on pre-game mood. The sample of Australian softball umpires reported mood profiles that were significantly different from non-athlete mood norms. As hypothesised (H1), participants reported more positive mood profiles than the general population, with very low mean scores for depression, anger, fatigue, and confusion, indicating a link between participation in softball umpiring and positive mental health. Consistent with our hypothesis (H2), umpiring performance was shown to be correctly classified from mood scores with 75% accuracy. Low scores for tension were particularly predictive of umpiring performances being rated as a pass. Mood scores also varied according to the sex, age, position on the diamond, and accreditation level of the umpires involved in the study.

Umpiring decisions often conflict with the opinions of athletes and spectators, resulting in umpires experiencing derision and ridicule [26], abuse, and occasionally violence [27]. This suggests that managing anger responses is a factor in promoting good performance as an umpire. In the present sample, anger scores were uniformly low across the participants, with 99.5% of mood profiles having anger scores at or close to zero, suggestive of positive mental health. Overall, assessments of pre-game mood among softball umpires showed that participants tended to report either iceberg or submerged profiles with no reported inverse Everest, inverse iceberg, or shark fin profiles, which are proposed to reflect elevated risk of mental health issues [11]. Hence, no evidence of mental ill-health or risks to mental health was identified in any participant.

When umpiring performance was dichotomised into pass and fail categories, participants who failed reported higher pre-game tension scores. Tension is known to affect decision making due to the increase in cognitive load, and performance is expected to suffer if the individual’s interpretation of this tension is that it will debilitate their performance [42]. A conceptual model of mood and performance relationships in sports [43] emphasised the interactive effects of different mood dimensions on performance. Notably, the model proposed that tension has a negative effect on performance when symptoms of depression are experienced simultaneously, whereas in the absence of any depression symptoms, tension shows an inverted-U relationship with performance (i.e., tension is facilitative of performance up to an optimum point, and thereafter, further increases in tension are debilitative of performance). Applying this model to softball umpiring, given that some level of tension is both inevitable and desirable, the interaction of symptoms of tension and depressed mood, even mild symptoms such as temporary unhappiness, has the potential to debilitate umpiring performance. For example, umpires appointed to officiate teams that had previously treated them disparagingly, had very highly skilled participants, and/or had a history of angry confrontation may experience tension accompanied by a level of depressed mood that in combination produces poor umpiring performance. The current study could be extended in the future via a qualitative investigation to clarify the thought processes and considerations underlying the observed tension scores.

The sex of the participants explained 25.7% of the overall variance in mood scores. Typically, among athletes and non-athletes, males tend to report more positive mood profiles than females [31,44,45]. Counter to our hypothesis (H3), the opposite was found in our study, with significantly higher depression and lower vigour scores reported by males than by females. Given that this finding was derived from a small sample of umpires, the results may be anomalous. However, taking the results at face value, there may be a case for male umpires in particular to use evidence-based mood enhancement strategies in the pre-game period, such as listening to their favourite music or engaging in a vigorous warm-up routine [46]. It should be noted that the BRUMS assesses depressed mood rather than clinical depression, and both male and female participants reported depression scores that were significantly below the general population means, with 96.2% of the scores below the 48th percentile.

The age of the participant accounted for 25.8% of the variance in mood scores. Older participants, aged 51+ years, reported significantly lower scores for vigour and higher scores for depression than their younger colleagues. Age-related differences in BRUMS scores have been reported previously in the literature [31,45], although the present results do not align closely with those prior findings and contradict our hypothesis (H4). While it could be expected that younger participants would report higher vigour scores than participants in their 50s and 60s, in relation to depression scores, the results of a large-scale study involving nearly 16,000 participants reported that BRUMS depression scores tended to decline rather than increase among those in their 50s and beyond [31]. The present findings related to age may have been confounded by the sex of participants, given that the female participants in our sample were on average 16 years younger than the male participants, and the results for age closely mirrored the results for sex.

Regarding umpire position on the diamond, as hypothesised (H5), mean tension scores were significantly higher when the participant assumed the plate position than when the participant officiated in the field. This result was anticipated given that the number and significance of decisions made by plate umpires have a greater overall impact on game outcome than those of field umpires. The higher tension scores reported by plate umpires may possibly be facilitative of performance by helping participants maintain focus throughout a game in accordance with the theory of facilitative and debilitative anxiety [42]. However, applying the conceptual model of mood and performance relationships described earlier [43], the effect of tension on performance is influenced by the simultaneous presence or absence of symptoms of depressed mood. It should be noted that although tension was higher among participants performing as plate umpires than field umpires, the iceberg profile was reported by both groups, confirming that participants reported a mood profile associated with positive mental health and good performance regardless of their position on the diamond.

In relation to the umpiring accreditation level of participants, consistent with our hypothesis (H6), those at the higher accreditation levels reported significantly lower fatigue scores than those at the lower accreditation levels. Fatigue is known to impact decision making in sports by affecting attention and working memory, which are essential cognitive functions in fast-moving sports environments [47,48]. Participants in the study typically officiated two games daily at championships played over a 7-day period, remained at the ballpark for the majority of each day even when not umpiring, stayed in shared room accommodation (potentially affecting sleep quality and duration [49]), and officiated in up to 13 games of 62 to 172 min in duration. This situation is very different from weekend local association or state championship events that would be more familiar to lower accredited umpires. Local/state events are of shorter overall duration (up to three days if played over a long weekend), and national games are not bound by maximum game time limits as are local and state games. In addition, with some exceptions, umpires generally have the convenience of sleeping in their own beds when officiating local and state games. As a dimension of mood, fatigue has been shown to adversely affect performance in sports [28,29]. In softball, fatigue may negatively impact an umpire’s ability to read the game effectively, move to optimal positions to make judgements, and correctly recall rules during pressure situations. Therefore, developing strategies to effectively manage fatigue may be a factor that contributes to softball umpires achieving higher levels of accreditation, highlighting a potential need for umpire training in recovery strategies.

We acknowledge some limitations of our study. Firstly, the relatively small number of umpires who participated in the study restricts the broad generalisation of the findings. The limited sample size is reflective (a) of softball being a minor sport in Australia with only a small number of umpires officiating at the national championships and (b) the cancellation of the softball national championships due to COVID-19 in the year following the collection of data preventing an opportunity for additional data collection. Although these two factors combined to limit the size of the participant group, we contend that the significant findings we have reported provide a valuable point of reference for future mood-related research with sport officials, especially softball umpires. Secondly, our study investigated interactor-style officials, and the results may not be relevant to monitor-style or reactor-style officials due to differences in the level of physical activity involved and the type and number of interactions with the athletes for each category of official. Future studies could involve participants from all three classification styles to increase the generalisability of findings.

The present findings may help to inform efforts by Softball Australia to improve umpire performance by developing and including psychological interventions in programs run by umpire trainers, mentors, and sport psychologists. For example, training clinics and pre-assessment events attended by umpires-in-training in preparation for presenting for assessment to the next accreditation level [50] offer the opportunity to provide group training in evidenced-based mood regulation strategies [46]. Umpires are already encouraged to work with mentors, and such relationships offer developing umpires one-on-one tutoring and support. Lastly, national championships, at which the umpires operate in a team-based environment, could provide opportunities for sport psychologists to engage in more advanced psychological interventions designed to help umpires improve their on-diamond performance. Softball is a relatively small sport in Australia, although that is not the case in other countries. For example, softball is a popular sport in the USA with more than 8 million participants in 2021 [51]. It is anticipated that the results of this study and the interventions proposed would be replicable in countries with a large softball community.

## 5. Conclusions

Several conclusions can be drawn from the results of the present study. Firstly, softball umpires reported mood profiles that were more positive than the general population and were associated with good mental health. Secondly, mood was predictive of umpire performance, with 75% of performances being correctly classified as pass or fail from pre-game mood scores. Thirdly, group differences in mood were found, with sex explaining 25.7% of the variance in mood scores, age group explaining 31.8%, position on the diamond explaining 10.5%, and accreditation level explaining 14.3%

## Figures and Tables

**Table 1:** Umpire mood scores (*N* = 185) and comparison with non-athlete norms.

Dimension	Minimum	Maximum	Mean	SD	SE	t	*p*	d
Tension	41	85	46.28	5.89	0.43	5.05	<0.001	−0.63
Depression	43	59	44.09	2.04	0.15	8.04	<0.001	−2.90
Anger	43	56	43.14	1.04	0.08	9.33	<0.001	−6.60
Vigour	30	72	51.55	7.70	0.57	2.10	0.040	0.20
Fatigue	36	65	40.36	4.92	0.36	13.09	<0.001	−1.96
Confusion	42	55	42.48	1.82	0.13	10.23	<0.001	−4.13

**Table 2:** MANOVA comparing mood scores by umpiring performance.

	Fail (n = 30)			Pass (n = 155)					
Dimension	Mean	SD	SE	Mean	SD	SE	F	*p*	Partial η^2^
Tension	49.87	8.93	1.60	45.58	4.86	0.40	14.21	<0.001	0.072
Depression	44.77	3.29	0.60	43.96	1.69	0.14	3.97	0.048	0.021
Anger	43.20	0.76	0.14	43.12	1.10	0.09	0.14	0.712	0.001
Vigour	50.97	7.11	1.30	51.66	7.85	0.63	0.20	0.652	0.001
Fatigue	40.03	3.31	0.60	40.42	5.20	0.42	0.15	0.696	0.001
Confusion	43.03	2.27	0.41	42.37	1.71	0.14	3.40	0.067	0.018

**Table 3:** MANOVA comparing mood scores of female and male umpires.

	Females n = 69			Males n = 116					
Dimension	Mean	SD	SE	Mean	SD	SE	F	*p*	Partial η^2^
Tension	47.72	8.27	1.00	45.41	3.65	0.34	6.85	0.010	0.036
Depression	43.16	0.96	0.12	44.65	2.30	0.21	26.07	<0.001	0.125
Anger	43.00	0.00	0.00	43.22	1.32	0.12	1.84	0.176	0.010
Vigour	54.70	6.31	0.76	49.68	7.90	0.73	20.14	0.001	0.099
Fatigue	39.38	4.08	0.49	40.94	5.31	0.49	4.42	0.037	0.024
Confusion	42.39	1.25	0.15	42.53	2.09	0.19	0.24	0.628	0.001

**Table 4:** MANOVA comparing mood scores by umpire age.

	23–50 Years (n = 82)			51+ Years (n = 103)					
Dimension	Mean	SD	SE	Mean	SD	SE	F	*p*	Partial η^2^
Tension	47.46	7.56	0.84	45.33	3.92	0.39	6.13	0.014	0.032
Depression	43.37	0.73	0.08	44.67	2.52	0.25	20.60	<0.001	0.101
Anger	43.00	0.00	0.00	43.24	1.40	0.14	2.47	0.117	0.013
Vigour	54.88	6.44	0.71	48.90	7.66	0.76	31.93	<0.001	0.149
Fatigue	40.76	5.00	0.55	40.04	4.89	0.48	0.96	0.328	0.005
Confusion	42.59	1.75	0.19	42.39	1.88	0.19	0.53	0.466	0.003

**Table 5:** MANOVA comparing mood scores by field position.

	Plate (n = 59)			Field (n = 126)					
Dimension	Mean	SD	SE	Mean	SD	SE	F	*p*	Partial η^2^
Tension	48.41	7.66	1.00	45.28	4.57	0.41	11.97	<0.001	0.061
Depression	44.12	2.31	0.30	44.08	1.91	0.17	0.02	0.903	0.000
Anger	43.00	0.00	0.00	43.20	1.27	0.11	1.45	0.231	0.008
Vigour	52.27	7.89	0.03	51.21	7.65	0.68	0.75	0.387	0.004
Fatigue	39.25	3.81	0.50	40.87	5.32	0.47	4.40	0.037	0.023
Confusion	42.47	1.65	0.22	42.48	1.90	0.17	0.00	0.996	0.000

**Table 6:** MANOVA comparing mood scores by umpire accreditation level.

	Level 4–6 (n = 164)			Level 7–8 (n = 21)					
Dimension	Mean	SD	SE	Mean	SD	SE	F	*p*	Partial η^2^
Tension	46.73	6.10	0.48	42.71	1.55	0.34	9.00	0.003	0.047
Depression	44.12	2.10	0.16	43.86	1.49	0.33	0.31	0.577	0.002
Anger	43.13	1.09	0.09	43.14	0.66	0.14	0.00	0.971	0.000
Vigour	51.17	7.92	0.62	54.52	5.22	1.14	3.56	0.061	0.019
Fatigue	40.85	5.03	0.39	36.52	0.51	0.11	15.40	<0.001	0.078
Confusion	42.54	1.82	0.15	42.00	0.00	0.00	1.62	0.204	0.009

## Data Availability

Data are available from the first author on request.
